# Prevalence, Occurrence, and Characteristics of Supernumerary Teeth Among the Saudi Arabian Population Using Panoramic Radiographs

**DOI:** 10.3390/diagnostics14222542

**Published:** 2024-11-13

**Authors:** Sreekanth Kumar Mallineni, Sami Aldhuwayhi, Yahya Deeban, Khalid Saud Almutairi, Sultan Nawasir Alhabrdi, Mohammad Abdulaziz Almidaj, Bader Abdullah Alrumi, Abdurrahman Salman Assalman, Angel Mary Joseph, Amar Ashok Thakare, Mohammed Ziauddeen Mustafa

**Affiliations:** 1 Pediatric Dentistry, Dr. Sulaiman Al Habib Medical Group, Riyadh 14212, Saudi Arabia; 2 Division for Globalization Initiative, Liaison Center for Innovative Dentistry Graduate School of Dentistry, Tohoku University, Sendai 980-8575, Japan; 3 Center for Global Health Research, Saveetha Institute of Medical and Technical Sciences, Saveetha University, Chennai 602105, Tamil Nadu, India; 4 Department of Restorative Dentistry and Prosthodontics, College of Dentistry, Majmaah University, Al Majmaah 11952, Saudi Arabia; 5 Dental Department, Style Clinic, Riyadh 13313, Saudi Arabia; 6 Dental Department, Al-Zulfi General Hospital, Alzulfi, Riyadh 15972, Saudi Arabia; 7 Dental Department, Al-Artawiyah General Hospital, Al-Artawiyah, Riyadh 15736, Saudi Arabia

**Keywords:** supernumerary teeth, mesiodens, hyperdontia, prevalence, midline diastema, panoramic radiograph

## Abstract

Background: Supernumerary teeth numerical anomalies and the early diagnosis of supernumerary teeth is very important to avoid potential complications. The study aim was to determine the prevalence, occurrence, and characteristics of supernumerary teeth among the Arabian population. Methods: A retrospective radiographic study was performed using panoramic radiographs of patients attending a teaching hospital from January 2018 to December 2020. Only healthy patients with clear radiographs were included in the study, and patients with syndromes, cleft lip, and palate, and unclear radiographs were excluded from the study. The details include the patient’s age and gender, supernumerary tooth number, location, orientation, and position. Only a single examiner was involved in the data collection and analysis. Results: Overall, 38 (2%) patients were observed with 47 supernumerary teeth. Among them, 76% were males and 24% were females, with a mean age of 16.1 ± 9.7 years. Mesiodens (87%) are the common type of supernumerary tooth, and the majority of the supernumerary teeth were impacted (66%). The majority of the patients presented with a single supernumerary tooth, while 24% of the patients presented with two supernumerary teeth. Sixty percent of the supernumerary teeth were conical in morphology, followed by a tuberculate morphology. In the study population, most of the supernumerary teeth were normal in orientation. Conclusions: The prevalence of supernumerary teeth was 2%. Among them, the majority were observed at the anterior region of the maxillary arch with a conical shape of normal orientation. The gender-based comparison of location, orientation, morphology, eruption, and number of supernumerary teeth showed male predilection.

## 1. Introduction

Developmental anomalies of teeth and the surrounding structures are noticeable aberrations from the usual exhibition of both primary and permanent dentition. This category comprises an extensive array of disorders involving alterations in the quantity, shape, eruption time, type, location, and measurement of teeth [1,2]. The presence of such anomalies can obscure any dental treatment, especially in cases of root canal treatment, tooth extraction, etc., and might persuade malocclusion and functional and aesthetic complications [3]. Among these anomalies, numerical anomalies such as supernumerary teeth, hypodontia, and hypo-hyperdontia have significance in dental abnormalities [4,5]. Supernumerary teeth are defined as any tooth or odontogenic structure that is formed from a tooth germ in excess of the usual number for any given region of the dental arch [6]. This number difference can show up in one or more forms, on one side or both sides of the alveolar arches, and it is more likely to happen in the premaxillary region [7,8,9,10].

Supernumerary teeth are extra teeth to the normal complement; this is also called hyperdontia or third dentition [10,11,12]. Supernumerary teeth are more common in males than in females [4,5,7,13]. Atavism, the dichotomy theory, genetics, local trauma, and hyperactivity of the dental lamina are some of the causes that have been shown to lead to the development of extra teeth [13,14,15]. However, there is no globally accepted theory for the formation of supernumerary teeth [15]. Further, Brook et al. [16] opined that both environmental and genetic factors play an important role in the formation of supernumerary teeth. Supernumerary teeth can occur in single, double, and multiple ways; multiple supernumerary teeth are very common in patients with cleft lip and/or palate and cleidocranial dysplasia [17,18]. Syndromes can be linked to having extra teeth, and this number problem that does not happen with a syndrome has been written about a lot [19,20]. There are different types of supernumerary teeth based on their location (mesiodens, para-premolar, para-molar, and distomolar), morphology (tuberculate, conical, supplemental, and odontoma), orientation (vertical or normal, horizontal, and inverted), and position (buccal, palatal, and transverse) [21,22].

This number difference is linked to numerous problems in the developing teeth, such as midline diastema, delayed eruption, rotation, impaction, crowding, spreading, and dentigerous cysts. Therefore, early diagnosis and management of supernumerary teeth are very important to avoid such complications. In Saudi Arabia, the reported prevalence of supernumerary teeth ranges from 0.3 to 15.6% [23,24,25,26,27,28,29,30,31,32,33,34,35], and mesiodens have been reported to be a very common type of supernumerary teeth (Table 1). A few authors also reported the prevalence of dental anomalies; however, a couple of the studies involved the other nationalities along with Saudi nationalities and a few studies were not clear about their exclusion criteria [36,37]. None of the reported studies discussed the prevalence, occurrence, or characteristics of supernumerary teeth among the Arab population. Therefore, there is a need for the study to establish the common types and characteristics of supernumerary teeth. Nonetheless, the aim of this study was to establish the prevalence, occurrence, and characteristics of supernumerary teeth among the Saudi Arabian population.

## 2. Methods

Ethical approval was acquired by the Deanship of Scientific Research at Majmaah University in Al-Majmaah, Saudi Arabia, with the Institutional Review Board (IRB) number MUREC.May.06/COM-2020/29-1. This study was conducted in complete conformity with the World Medical Association Declaration of Helsinki. A retrospective radiographic study was performed using panoramic radiographs of patients attending a teaching hospital, the College of Dentistry, Al-Zulfi, Saudi Arabia, from January 2018 to December 2020. Saudi nationals, patients with clear panoramic radiographs, individuals who were visited for the first time, and individuals who had never had a history of extraction are the eligibility requirements for participation in the study. Patients with medically compromised syndromes, cleft lip and/or palate, unclear panoramic radiographs, and non-Saudi nationality were excluded from the study. All the panoramic radiographs were taken using the Kodak 8000C digital panoramic and cephalometric systems (Carestream Health, Inc., New York, NY, USA). All the radiographs were taken using standardized exposure parameters, adhering to all required radiation safety measures. The very first digital panoramic radiographs are for data collection purposes. None of the patients’ personally identifying information, such as their names and identification numbers, was involved in the data collection. A strict adherence to confidentiality was maintained, and every document was scrutinized to eliminate any previous instances of tooth extraction. The patient’s age, gender, median number of teeth, area, orientation, and position of the supernumerary teeth were all included in the data collection process. The comparison looked at gender (male and female), number (single, double, and more than two), orientation (transverse, normal, and inverted), position (impacted and erupted), morphology (tuberculate (supplemental considered as tuberculate), and conical), and location (mesiodens, para-premolar, para-molar, and distomolar) [17,18]. In the present study, the error of the approach was determined by doing the evaluations of dental anomalies (supernumerary and other dental anomalies) from twenty panoramic radiographs at regular intervals of two weeks. This was responsible for conducting all of the investigations. It was determined through the use of Kappa statistics whether or not the determination of each dental abnormality could be considered reliable. It was found that the initial and second evaluations produced a Kappa score of 0.9, and these radiographs were not included in the final study. The data collected was comprehensively analyzed using Excel 2000, a component of Microsoft Office designed in the United States (Microsoft Corporation, Washington, DC, USA). Frequencies and percentages were calculated to record the location, orientation, morphology, eruption, and number. The gender-based comparison was made based on number, dentition, arch, tooth type, tooth region, morphology, orientation, and eruption. The Chi square test used gender-based comparison. Data were analyzed using the Statistical Package for the Social Sciences (SPSS) version 21.0 (IBM Corp., Armonk, NY, USA) at a 5% significance level.

## 3. Results

**Figure 1:**
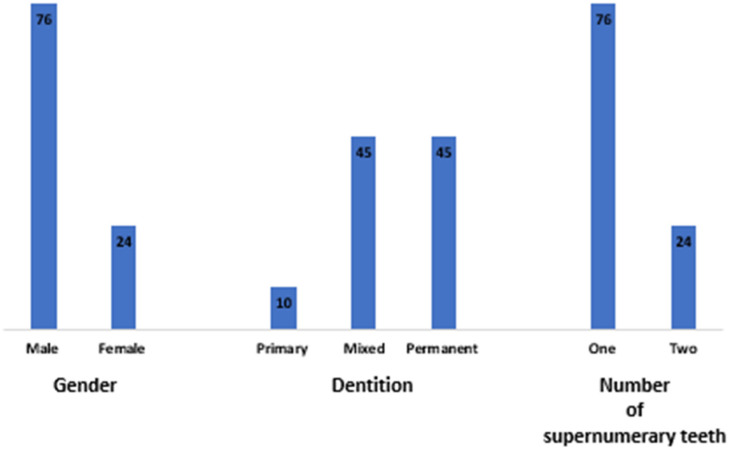
The distribution of supernumerary teeth in the study sample based on gender, dentition, and number of supernumerary teeth.

A total of 2438 panoramic radiographs were included in the study; among them, only 1901 were eligible for analysis. Thirty-eight patients (2%) of the total were found to have forty-seven supernumerary teeth with a mean age of 16.1 ± 9.7 years. The individuals included in this group consisted of 76% males and 24% females. Only 4% of the supernumerary teeth were observed in primary dentition. In comparison, 45% of supernumerary teeth in the study population reported mixed and permanent dentition. Seventy-four percent of the patients presented with a single supernumerary tooth, whereas twenty-four percent of the patients presented with two supernumerary teeth (Figure 1).

**Figure 2:**
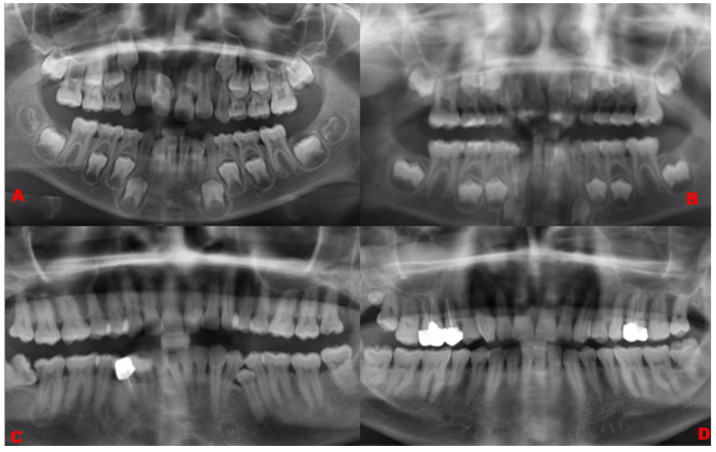
Cropped panoramic radiographs illustrating (**A**) inverted conical mesiodens, (**B**) tuberculate normally oriented mesiodens, (**C**) para-premolars, and (**D**) distomolar.

**Figure 3:**
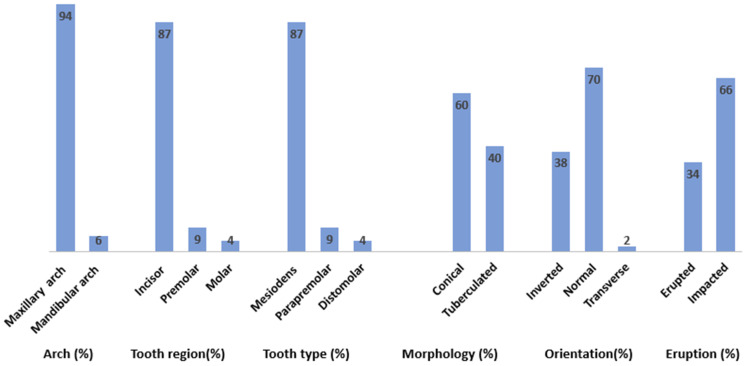
Distribution of supernumerary teeth based on arch type, tooth region, tooth type, morphology, orientation, and eruption of supernumerary teeth in the study population.

Mesiodens are the most prevalent type of supernumerary tooth, accounting for 87% of the total number of supernumerary teeth. Distomolars and parapremolars, on the other hand, were identified at 4% and 9%, respectively (Figure 2). Sixty-six percent of the supernumerary teeth were impacted, while thirty-four percent of the teeth were erupted. Sixty percent of the supernumerary teeth were identified as conical, while forty percent of them were found to be tuberculated. Eighty percent of the supernumerary teeth were in the anterior region (incisor region) while 13% of the supernumerary teeth were evident in the posterior region (premolar (9%) and molar (4%)). The majority of the supernumerary teeth were observed in the maxillary arch (94%) in contrast with the mandibular arch (6%). Seventy percent of the supernumerary teeth were normal in orientation, while 28% of the supernumerary teeth were inverted, and one supernumerary tooth (2%) was transverse in orientation (Figure 3).

**Figure 4:**
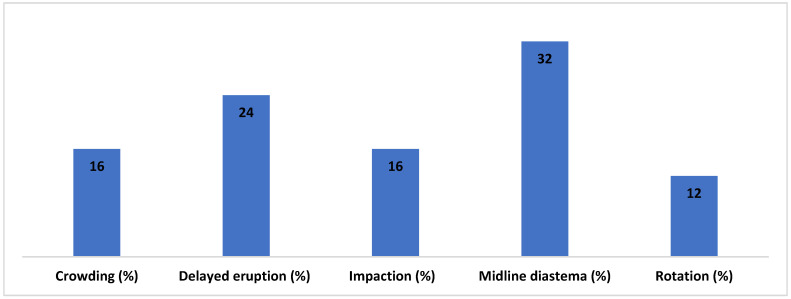
The complications associated with supernumerary teeth in the study sample.

Only 25 patients who had an excessive number of teeth reported complications, among them midline diastema, which accounted for 32% of the cases, and was a problem that was frequently related to supernumerary teeth in the population that was studied. Although patients with supernumerary teeth were more likely to have crowding, delayed eruptions, rotations, impactions, and rotations, respectively, were found in 16%, 24%, 16%, and 12% of patients (Figure 4).

The gender-based comparison was made based on number, dentition, arch, tooth type, tooth region, morphology, orientation, and eruption as illustrated in Figure 4. Males exhibited the majority of supernumerary teeth in mixed and permanent dentition, while females exhibited the majority of supernumerary teeth in primary dentition. The findings showed no significant difference (*p* > 0.05).

**Figure 5:**
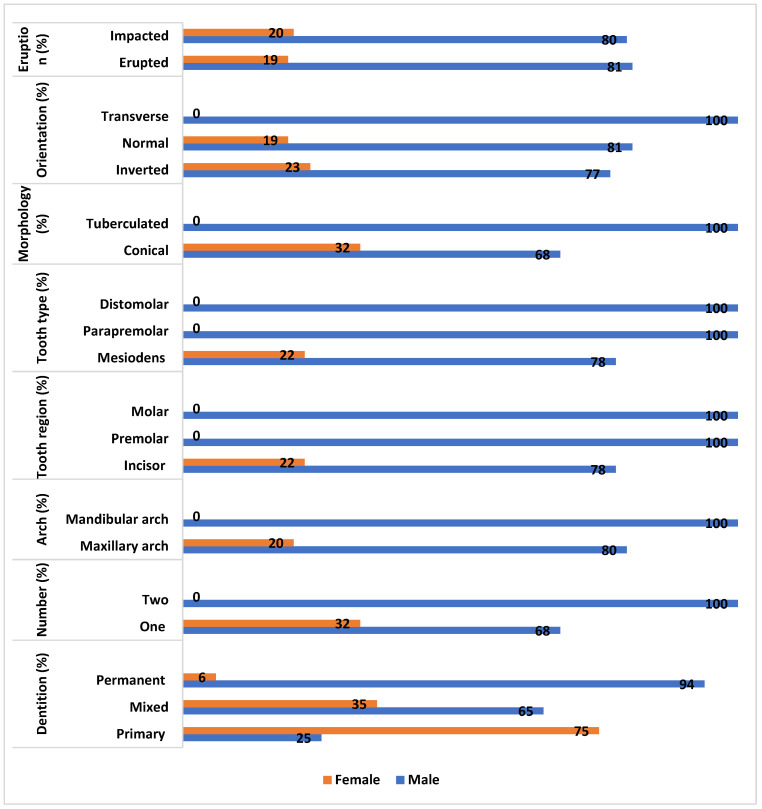
The gender-based comparison of supernumerary teeth is based on dentition, number, arch, tooth region, tooth type, orientation, morphology, and eruption.

Figure 5 summarizes the gender-based comparison of supernumerary teeth is based on dentition, number, arch, tooth region, tooth type, orientation, morphology, and eruption among the study population. The majority of the single supernumerary tooth observations were found in males (68%) and all the two supernumerary teeth observations were evident in males (*p* > 0.05). All the supernumerary teeth in the mandibular arch were found in males, while 80% were reported in males and 20% were found in males (*p* > 0.05). All the distomolars and para premolars were observed in males, while 78% of the mesiodens were observed in males and 22% of the mesiodens were observed in females (*p* > 0.05). All the supernumerary teeth in the premolar region and molar region were observed in males while 78% of the supernumerary teeth in males were observed in the incisor region and 22% in females (*p* > 0.05). Males accounted for 68% of the observed supernumerary teeth, while females accounted for 32%, and males accounted for 100% of the tuberculate supernumerary teeth (*p* > 0.05). Males accounted for all the transverse supernumerary teeth observed. Eighty-one percent of supernumerary teeth were normally oriented in males, while 77% of the inverted supernumerary teeth were reported in males (*p* > 0.05). All but 81% of supernumerary teeth in males were erupted, while 19% of supernumerary teeth in females were erupted. In males, 80% of the supernumerary teeth were impacted, while 20% were in females (*p* > 0.05).

## 4. Discussion

A comprehensive clinical examination, along with certain radiographic techniques, is crucial for accurately assessing the occurrence of supernumerary teeth. However, this importance is often overlooked or undervalued. The presence of several inconsistencies in the prevalence statistics raises doubts about the reliability of the existing data on supernumerary teeth. Some of the problems that can happen because of developmental problems in primary dentition are bad bites, cavities, poor aesthetics, issues with the eruption of the next tooth, and problems during endodontic or surgical procedures on the affected teeth. These issues may present challenges, ultimately resulting in irreversible damage. Typically, these abnormalities are disregarded, neglected, or go unreported during regular medical procedures and are infrequently reported by patients as their main concerns. The prevalence and distribution of these anomalies will assist dental faculty and experts in early identification and recognition, as well as in providing complete treatment for predominant dental malformations.

The prevalence of supernumerary teeth has not been reported in the Zulfi region of Saudi Arabia. There are only twelve studies reported from Saudi Arabia on the prevalence of supernumerary teeth, which ranges from 0.3% to 15.6%. Among the reported studies, two each came from Abha [28,31], Jazan [27,35], Jeddah [24,25], Dammam [32,33], and Gizan [23], Taif [34], Makkah [29], and Riyadh [30]. The present study was reported by a faculty of dentistry in Zulfi, Majmaah University, AlMajmaah Saudi Arabia. The present study found that 2% of the study sample was supernumerary. All the study samples published in Saudi Arabia were taken from the teaching hospitals in representative areas; similarly, the present sample was also taken from a teaching hospital at Majmaah University in Saudi Arabia. In the present study, the mean age was 16.1 ± 9.7 years, and the study sample involved an age range of five years to thirty-eight years with male predilection. The findings are in agreement with most of the studies reported in the literature and in Saudi Arabia. However, a study by Riyadh found female predilection of supernumerary teeth in the sample size, similarly. The sample involved 299 [30] to 2481 [34]. However, the present study sample size was 1901 of 2438 panoramic radiographs from the Zulfi region. The diagnostic tool used for the identification of supernumerary teeth plays a vital role in the diagnosis of supernumerary teeth. The previous studies used clinical records, study casts, and panoramic radiographs. It has been reported that there are various techniques used to identify dental abnormalities [38]. Nevertheless, Anthonappa et al. [39] opined that the identification of supernumerary teeth on panoramic radiographs required refined dental training. Among the prior studies from Saudi Arabia, a few researchers used only panoramic radiographs [25,26,27,29,30,32,35], and some of the investigators used clinical and radiographic records [23,24,28,34], whereas a recent study [31] from the Abha region used panoramic radiographs and periapical films. In the present study, the authors used panoramic radiographs for the data collection.

Supernumerary teeth can occur single or multiple times in any given region of the jaw [40,41,42,43,44]. According to a Brazilian study [41], 92.5% of the cases had a single supernumerary tooth, while the presence of two supernumerary teeth was detected in 7.5%. Similarly, a Jordanian study [42] found that 77% had single supernumerary teeth, 18.4% had two supernumerary teeth, and 4.65 had three supernumerary teeth. An Indian study found [43] that 79.1% have a single supernumerary tooth and 21.9% have two supernumerary teeth. A Turkish study [44] found that 78.8% had a single supernumerary tooth, whereas 20.4% had two supernumerary teeth. A Spanish study [45] also reported that 67.5% have single supernumerary teeth, whereas 25.5% have two supernumerary teeth. Similarly, in the present study, seventy-six percent of the patients presented with a single supernumerary tooth, whereas twenty-four percent presented with two supernumerary teeth. None of the prior published studies from Saudi Arabia reported on the number and distribution of supernumerary teeth. Some of the studies [46,47] reported the occurrence of multiple supernumerary teeth in healthy individuals. However, in the present study, there was no evidence of multiple supernumerary teeth.

Based on the published literature, mesiodens are a commonly observed supernumerary tooth type [41,42,43,44,45,46,47,48,49,50]. Similarly, in the present study, mesiodens are the most prevalent type of supernumerary tooth, accounting for 87% of the total supernumerary teeth observed. Distomolars and parapremolars, on the other hand, were identified in 4% and 9%, respectively. The majority of the studies from Saudi Arabia reported that mesiodens are a common type of supernumerary [26,28,29,32,34]. A study from Riyadh found distomolars to be the most common supernumerary type, but it only included orthodontic patients [30]. Another study from Jizan reported that parapremolars are commonly occurring in supernumerary tooth types in their study sample [25]. In agreement with the majority of the studies published in the literature, the present study found that 87% of the supernumerary teeth are mesiodens. Paramolars were not evident in the study population of the present study. A Jordanian [42] study found 0.5% in the molar region; in contrast, in the present study, the authors observed 4% of supernumerary teeth in the molar region. Kara et al. [44] evaluated supernumerary molars among the 104,902 Turkish population and found that in 0.33% of occurrences among them, 63% were distomolars and 37% were paramolars. Nonetheless, in the present study, none of the patients were observed with paramolars, and only 4% were reported with distomolars. On the other hand, a Brazilian study [41] found that none of the study population reported either paramolars or distomolars.

The majority (52.1%) of the supernumerary teeth were reported to have a normal orientation, while 2.5% of them were inverted, 35.9% were horizontal in orientation, and 9.4% of them had a horizontal orientation in a Turkish study [44]. These findings are not comparable with the present study since the Turkish study [44] only evaluated the supernumerary molars. The majority of the published studies reported that supernumerary teeth are inverted in orientation [45,46,47,48,49,50]. An Indian study [51] found that 68.5% of supernumerary teeth had a normal orientation and 24.1% had an inverted orientation. Inversely, a Japanese study [52] reported that 67% of inverted supernumerary teeth were normally oriented, 27% were normally oriented, and 6% were transversely oriented. Another Indian study [53] also reported that the majority (95.06%) of the supernumerary teeth were normally oriented, while 4.94% were inverted in orientation. A Hong Kong study [47] and a Turkish study [54] also found the majority of the supernumerary teeth in their studies to be normal in orientation. A Swedish study [55] reported that 51.3% of supernumerary teeth are normally oriented; however, 9.4% were horizontal and 39.3% were inverted in orientation. In the present study, 72% of the supernumerary teeth in the study were found to be normal in orientation. Whereas 26% of supernumerary teeth were inverted in orientation and 2% of them were with transverse orientation. None of the reported studies from Saudi Arabia evaluated the orientation of supernumerary teeth in their study population.

The conical form of the supernumerary tooth was the most prevalent, which is consistent with the published literature [16,47,48]. Sixty percent of the supernumerary teeth were identified as conical in shape, while forty percent of them were found to be tuberculated in the present study. A few researchers from India [46] and Korea [56], reported a higher percentage of conical-shaped supernumerary teeth. Inversely, a few of the researchers, including a Turkish study [54], a Swedish study [55], and a Brazilian study [41] reported lower percentages compared to the presented study. A Swedish study [55] found that the majority of the supernumerary teeth are conical (55.5%) in morphology. On the other hand, a Turkish study found that 47.3% of their cohort had conical supernumerary teeth, followed by 39.9% tuberculate and 12.8% with supplemental morphology. However, the authors of the study only included supernumerary molars, and these findings are comparable to those of the present study. An Indian study [46] reported that 79.4% of their cohort had conical-shaped supernumerary teeth, 9.75% had tuberculate, and 7.31% had the supplemental type. The Nepalese study [57] found that 58.1% of the supernumerary teeth had conical morphology, and tuberculate and supplemental teeth were 30.9% and 10.9%, respectively. A Chinese CBCT study [58] reported that 83.5% of the supernumerary teeth were conical in shape, followed by tuberculate. In the present study, conical and peg-shaped teeth were considered in the same category study and supplemental teeth were also considered as tuberculate. A Swedish study [55] evaluated conical and peg-shaped supernumerary teeth separately and a three-dimensional study from China [58] reported conical and peg-shaped teeth in the same category. In the present study, panoramic radiographs were used for the analysis, nonetheless, and the findings were not comparable with the Chinese study [58].

Sixty-six percent of the supernumerary teeth were impacted, while thirty-four percent of the teeth were erupted. Among 38 individuals with supernumerary teeth, 17 patients had (48.6%) supernumerary teeth that had erupted into the oral cavity. Twelve patients (34.3%) had supernumerary teeth that were impacted and the remaining six patients (17.1%) presented with both erupted as well as impacted teeth. The supernumerary teeth cause various problems including conditions from midline diastema to cystic formation [58,59,60,61]. Merely 25 patients who had an excessive number of teeth reported complications, among them midline diastema, which accounted for 32% of the cases. This was a problem that was frequently related to supernumerary teeth in the population that was studied. Although patients with supernumerary teeth were more likely to experience crowding, delayed eruptions, rotations, impactions, and rotations, respectively, 16%, 24%, 16%, and 12% of patients reported each of these complications. A Hongkong [47] study reported that 0.5% of supernumerary teeth were associated with dentigerous cysts; on the other hand, a Japanese study [60] reported that 2.5% of supernumerary teeth were associated with cystic formation. However, in the present study, none of the supernumerary teeth were associated with cystic formation.

This study also investigated the gender-based comparison of supernumerary teeth among the Saudi Arabian population. Most studies only concentrate on gender-based distribution worldwide. Almost all of the published reports observed supernumerary teeth as more common in males than females [41,42,43,47,48,49,50,51,52,62]. Similarly, among the studies published in Saudi Arabia, supernumerary teeth are more common in males compared to females [23,24,25,27,28,29,30,31,32,33,34,35]. However, Al-Jabaa and Aldrees [26] reported a female predilection of supernumerary teeth. All published reports in the literature, except a Brazilian study [62], evaluated supernumerary teeth based on tooth region and morphology. This study reported that conical, supplemental, and tuberculate supernumerary teeth were 2.75:1 (*p* < 0.05), 0.25:1 (*p* < 0.05), and 1.3:1 (*p* > 0.05), respectively. In the present study, the male and female ratios for conical and tuberculate were 2.1:1 (*p* < 0.05) and 1; no supplemental type was observed. In the case of the tooth region, they divided the midline, incisor, canine, and premolar, and the male/female ratio was 8:1 (*p* < 0.05), 0.3:1 (*p* < 0.05), 0.5:1 (*p* > 0.05), and 4:1 (*p* < 0.05). However, in the present study, we divided based on the previous classifications [4,16] of incisor, premolar, and molar, and the male/female ratio was 3.5:1 (*p* < 0.05), 4, and 2, respectively. Nonetheless, there are studies on the prevalence of specific dental anomalies, such as shape or size. Prior research has shown that the prevalence of various dental abnormalities varies considerably across different populations. No published studies from Saudi Arabia have detailed the occurrence and characteristics of supernumerary teeth in their samples [23,24,25,26,27,28,29,30,31,32,33,34,35]. None of the published studies [23,24,25,26,27,28,29,30,31,32,33,34,35] on these topics from Saudi Arabia has not reported on the distribution or prevalence of developmental defects in permanent dentition. Racial disparities, inconsistent or different diagnostic criteria, and different sample methods were the primary causes of this discrepancy in findings [44,46,62,63].

Limitations include the fact that only supernumerary teeth were taken for analysis. The sample (1901 of 2438 panoramic radiographs) consists of the entire group of patients visiting a teaching hospital in Al Zulfi during the study period and the entire sample from the database was not included in the study. The panoramic radiographs were used for the analysis of the prevalence, occurrence, and characteristics of the supernumerary teeth. These panoramic radiographs only provide a two-dimensional image of a three-dimensional structure. The 3D images would have provided accurate information, and thus this can be considered as a limitation of the study. The dental anomalies associated with supernumerary teeth have not been included in the study, and this also can be a limitation of the study. The subjects with all the dentition were involved in the study, and the dentition-based analysis was not made. To the best of the authors’ knowledge, this is the only study discussing the prevalence, occurrence, and characteristics of supernumerary teeth from Saudi Arabia.

## 5. Conclusions

The prevalence of supernumerary teeth in the Saudi Arabian population was 2%. The findings of the present study within the limitations revealed that the majority of the supernumerary teeth were conical, occurred in the premaxillary region with normal orientation, and were erupted. The gender-based comparison of location, orientation, morphology, eruption, and number of supernumerary teeth showed male predilection. A thorough knowledge of supernumerary teeth will help in the diagnosis and proper treatment planning of these conditions.

## Figures and Tables

**Table 1:** The reported prevalence of supernumerary teeth among the Saudi Arabian population.

Author	Year	Sample	Region	Diagnostics Tool	Age	Prevalence	Male	Female	Common Type
Salem [23]	1989	2393	Gizan	Clinical and radiographs	4–12	0.5	2	1	-
Ghaznawi et al. [24]	1999	1010	Jeddah	Clinical and radiographs	12–40	1.19	1.69	0.63	-
Afify and Zawawi [25]	2012	878	Jeddah	Radiographs	12–30	0.3	2.5	1	-
Al-Jabaa and Aldrees [26]	2013	602	Riyadh	Radiographs	5.1	5	5	6	Mesiodens
Vani et al. [27]	2015	1000	Jazan	Radiographs	18–40	1	1.2	0.8	Parapremolars
Yassin [28]	2016	1252	Abha	Clinical and radiographs	5–12	3.5	3.5	2	Mesiodens
Al-Halal et al. [29]	2017	1019	Makkah	Radiographs		2.3	2.66	1	Mesiodens
Alswayyed et al. [30]	2018	299	Riyadh	Radiographs	14–30	0.3	1		Distomolar
Zakirulla et al. [31]	2019	1350	Abha	Radiographs	5–15	5.2	4.6	2.5	Mesiodens
ALHumaid et al. [32]	2021	1189	Dammam	Radiographs	7–65	1.8	2.5	2	-
Bakhurji et al. [33]	2021	2226	Dammam	Radiographs	6–18	0.5	5	4	Mesiodens
Aljuai et al. [34]	2022	2481	Taif	Clinical and radiographs	>18	15.6	8	7.4	-
Renugalakshmi et al. [35]	2023	1442	Jazan	Radiographs	5–17	1.24	1.43	1	-

## Data Availability

Data will be available upon request to the corresponding authors.
